# Pennsylvania Considering Sterilization of Defectives

**Published:** 1911-07

**Authors:** 


### Pennsylvania Considering Sterilization of Defectives.

A bill for sterilization of idiots, feeble-minded and insane persons in State institutions has been presented in the Pennsylvania House by Mr. Freeman, Lebanon. The bill was drawn by Director of Public Health and Charities Neff, of Philadelphia, and Secretaries Wharton and Woodbury, of the State Board of Charities and Committee on Lunacy. The bill provides that a commission of physicians, surgeons and neurologists shall pass on the patient's mental state and then report to the managers of the institution, who shall then determine whether he or she shall be operated on. The report must then go to the court of the county where the hospital is located for approval. In case of approval the operation is to be performed.—*Medical Fortnightly.*

---

Many, if not most, of the cases of shoulder pain variously diagnosed as circumflex neuritis, rheumatism, etc., are instances of subacromial or subcoracoid bursitis. With involvement of the former bursa there is usually tenderness just beyond the acromion, usually somewhat anteriorly; in the latter there is marked tenderness just external to the coracoid tip. Pain or rotation of the arm may be present in either; it is most constant in subcoracoid bursitis.—*American Journal of Surgery.*

